# Editorial: Genome-wide analyses of *Pectobacterium* and *Dickeya* species, volume II

**DOI:** 10.3389/fpls.2023.1224293

**Published:** 2023-06-02

**Authors:** Robert Czajkowski, Mohammad Arif, Toni Chapman

**Affiliations:** ^1^ Laboratory of Biologically Active Compounds, Intercollegiate Faculty of Biotechnology Univesity of Gdansk (UG) and Medical University of Gdansk (MUG), University of Gdansk, Gdansk, Poland; ^2^ Department of Plant and Environmental Protection Sciences, University of Hawaii at Manoa, Honolulu, HI, United States; ^3^ Biosecurity and Food Safety, New South Wales (NSW) Department of Primary Industries, Elizabeth Macarthur Agricultural Institute (EMAI), Menangle, NSW, Australia

**Keywords:** soft rot *Pectobacteriaceae* (SRP), genomics, plant pathogens, plant-microbe interaction, control

Soft Rot *Pectobacteriaceae* (SRP), including *Pectobacterium* spp. and *Dickeya* spp., are Gram-negative phytopathogens with pectinolytic properties. This destructive group of phytobacteria pose a significant threat to global crop and ornamental plant production, causing extensive damage (Charkowski, 2018; van der Wolf et al., 2021a). At the moment, the *Dickeya* genus contains 12 species, including *D. dianthicola*, *D. dadantii*, *D. zeae*, *D. chrysanthemi*, *D. paradisiaca*, *D. solani*, *D. aquatica*, *D. fangzhongdai*, *D. poaceaphilia*, *D. lacustris*, *D. undicola*, and *D. oryzae* (Van Gijsegem et al., 2021). The genus *Pectobacterium* currently contains 20 species, including *P. actinidiae*, *P. aquaticum*, *P. aroidearum*, *P. atrosepticum*, *P. betavasculorum*, *P. brasiliense*, *P. cacticida*, *P. carotovorum*, *P. fontis*, *P. odoriferum*, *P. parmentieri*, *P. parvum*, *P. peruviense*, *P. polaris*, *P. polonicum*, *P. punjabense*, *P. quasiaquaticum*, *P. versatile*, *P. wasabiae*, and *P. zantedeschiae* (Van Gijsegem et al., 2021). All SRP species are characterized as necrotrophic pathogens capable of rapidly degrading plant tissue components upon which they feed (Toth and Birch, 2005).

The estimated global costs associated with SRP bacteria in agriculture are high and continuously rising (van der Wolf et al., 2021a). As SRP are globally distributed and known to infect a wide range of monocot and dicot plants across diverse climatic conditions, the estimated cost of their presence and activities in vegetable, fruit, and ornamental plant production can reach up to 100 million USD annually (Dupuis et al., 2021; Arif et al., 2022).

The success of SRP as pathogens relies on their ability to produce a diverse array of effectors, which play a crucial role in the colonization and establishment of infection in plants (van der Wolf et al., 2021b; Van Gijsegem et al., 2021). These effectors include plant cell wall-degrading enzymes (PCWDEs), lipopolysaccharides (LPS), extracellular polymeric substances (EPS), indigoidine, siderophores, type I-IV secretion systems, enterobacterial common antigen (ECA), necrosis-inducing protein (Nip), citrate uptake, ferredoxin-like protein (FerE) motility and adhesion to plant tissues (Reverchon et al., 2016; Arizala and Arif, 2019).

Although SRP bacteria have been extensively researched for over 50 years, studies targeting the global molecular background of virulence, host recognition, and niche adaptation of SRP bacteria to new hosts are limited. However, the recent advances in sequencing technologies combined with the rapidly decreasing sequencing costs have enabled targeting these critical research issues on a new scale.

In this Research Topic, four articles were published. Robic et al. study used transposon-sequencing to assess genes exclusively expressed in *Dickeya solani* during colonization and infection of potato roots, stems and tubers. They found 126 *D. solani* genes essential for the competitive colonization of potato tuber tissue, 207 genes crucial for disease progression in infected stems, and 83 genes necessary for the colonization of potato roots by the bacterium. In addition, several *D. solani* root-colonization-essential genes were encoding proteins involved in the utilization of organic and mineral nutrients and the synthesis of metabolites helping the bacteria to invade plant tissues from soil. The authors selected four genes: *bcsA*, *ddpA*, *apeH*, and *pstA*, and constructed their in-frame deletion mutants. The obtained mutants were virulent in stem assays. Still, they were impaired in colonizing potato roots. Such results indicate that depending on the nutrient availability, *D. solani* may exploit two distinct life strategies: oligotrophic on roots when the nutrient availability is limited and copiotrophic in nutrient-rich environments of macerated stem and tuber tissues.

In another article, Zhou et al. reported a new *Pectobacterium* species isolated from taro (*Colocasia esculenta*) and named it *P. colocasium*. Likewise, the authors showed for the first time that another *Pectobacterium* species, *P. aroidearum*, can infect taro and establish a successful infection in this plant under natural conditions. This is the first study describing new pathogens causing taro soft rot in China. Furthermore, Zhou et al. analyzed the interaction of *P. colocasium* LJ1 and *P. aroidearum* LJ2 in the development of disease symptoms in taro. They showed that even though both pathogens were present in the same plant, they neither expressed synergistic nor antagonistic interactions with each other. Comparative analyses of the genome sequences of LJ1 and LJ2 strains and known genomes of *Pectobacterium* species revealed the existence of unique pathogenicity-related features present in LJ1 and LJ2 strains, including the variation in the copy number and organization of type III, IV, and VI secretion systems and differential production of plant cell wall degrading enzymes. These results may shed light on the SRP effectors used by bacteria to infect various plant hosts as well as on the simultaneous co-infection of the plants with several *Pectobacterium* pathogens.

Zhang et al. analyzed the genomes of three *D. zeae* strains isolated from banana. The authors obtained high-quality and complete genome sequences of strains MS1, MS_2014, and MS_2018 isolated from symptomatic banana plants and determined their genomic and phenotypic diversity. Compared to the other representative *D. zeae* MS2 from banana, MS1, MS_2014, and MS_2018 strains were similar in most features, including the utilization of carbon and nitrogen sources, the general genomic features of GC content, and tRNA and rRNA genes. In addition, the three strains were also conserved in most virulence determinants. Contrarily, MS1, MS_2014, and MS_2018 strains expressed dissimilarities in flagellar gene clusters and clusters involved in the production of secondary metabolites, including bacteriocin and aryl polyene. Phylogenomic analysis of strains MS1, MS2 MS_2014, and MS_2018 confirmed the genomic divergence among *D. zeae* strains isolated from banana. Considering that these strains differed in their virulence, the study may help to predict the risk of spreading the new *D. zeae* variants in banana production sites and help to develop risk assessment and monitoring methods to minimize the impact of the pathogen.

In the last article, Liu et al. reviewed the available information about quorum sensing systems present in *Dickeya* spp. and how they collaborate in the pathogenic behavior of the bacteria during infection. The article of Liu et al. is by far one of the most recent and detailed analyses of the collective activity of SRP bacteria in the environment and its regulation. It can help to design new *Dickeya* spp.-control strategies based on interfering with the quorum sensing systems.

In conclusion, this Research Topic compiled research articles covering a wide range of Research Topics related to genome-wide analyses of SRP bacteria. The articles published in this Research Topic added valuable scientific knowledge to the existing studies on SRP pathogenicity, host range, adaptation, and control. In addition, the insights and findings presented in these articles will provide a valuable resource for future molecular studies on this agriculturally important group of bacterial pathogens.

## Author contributions

All authors listed have made a substantial, direct, and intellectual contribution to the work and approved it for publication.
